# The experiences of culturally and linguistically diverse family caregivers in utilising dementia services in Australia

**DOI:** 10.1186/1472-6963-13-427

**Published:** 2013-10-22

**Authors:** Lily Dongxia Xiao, Anita De Bellis, Lesley Habel, Helena Kyriazopoulos

**Affiliations:** 1 Flinders University, GPO Box 2100, Adelaide 5001, Australia; 2 Alzheimer’s Australia South Australia, 27 Conyngham Street, Glenside, SA 5065, Australia

**Keywords:** Dementia, Caregiver/caregiving, Community-based aged care, Dementia care services, Ethnicity

## Abstract

**Background:**

Older people from culturally and linguistically diverse groups are underrepresented in residential aged care but overrepresented in community aged care in Australia. However, little is known about culturally and linguistically diverse family caregivers in utilising dementia services in Australia because previous studies mainly focused on the majority cultural group. Experiences of caregivers from culturally and linguistically diverse groups who are eligible to utilise dementia services in Australia are needed in order to optimize the utilisation of dementia services for these caregivers.

**Methods:**

The aim of the study was to explore the experiences of family caregivers from Chinese, Greek, Italian and Vietnamese groups in utilising dementia services. Gadamer's philosophical hermeneutics was used to interpret the experiences of the participants. Focus group discussions and in-depth individual interviews were used to collect data. Data collection was conducted over a six month period in 2011. In total, 46 family caregivers who were caring for 39 persons with dementia participated.

**Results:**

Four themes were revealed: (1) negotiating services for the person with dementia; (2) the impact of acculturation on service utilisation; (3) the characteristics of satisfactory services; and (4) negative experiences in utilising services. The present study revealed that the participation of caregivers from culturally and linguistically diverse groups in planning and managing dementia services ranged markedly from limited participation to full participation.

**Conclusions:**

The findings of this study suggest that caregivers from culturally and linguistically diverse groups need to be fully prepared so they can participate in the utilisation of dementia services available to them in Australia.

## Background

Australia is one of a few developed countries in the world that relies heavily on immigration to maintain its population growth. Overseas-born older people comprise 35% of the population that is aged 65 years or over and of these, 61% are immigrants from non-English-speaking countries [1]. The prevalence of dementia in Australia is estimated to triple from 298,000 in 2011 to 891,400 in 2050. Among people with dementia in Australia, approximately 12.4% do not speak English at home. In 2010, 35,549 people with dementia were from culturally and linguistically diverse (CaLD) groups and this number is expected to reach 119,582 by 2050 [2,3]. Compared to the Australian-born group, CaLD groups used community aged care services relatively more frequently.

The current community aged care services in Australia are divided into low care services called community aged care packages (CACP) and high care programs that include the extended community aged care at home (EACH) and extended community aged care dementia (EACH-D) programs [3]. The EACH and EACH-D programs aim to provide frail and elderly patients who prefer to live at home with clinical nursing care and behavioural management from multidisciplinary teams of health professionals [4,5]. In addition, the National Respite for Caregivers Program (NRCP) assists the caregivers themselves [3]. The usage rates of CACP and EACH (including EACH-D) were 0.75% and 0.2% respectively in CaLD groups in 2012, which were higher than those in the Australian-born group described as 0.73% and 0.16% respectively [3]. The CaLD groups used NRCP as frequently as the Australian-born group. Despite this frequency of use, little is known about culturally and linguistically diverse family caregivers in utilising dementia services in Australia because previous studies mainly focused on the majority cultural group.

The current community aged care services are viewed as agency-centred, fragmented and inflexible [4,6,7]. This means that CaLD caregivers of people with dementia, who often have English as a second language (or do not speak English) and have limited health literacy, are likely to face enormous challenges in utilising dementia services. Indeed, several studies have revealed unmet dementia care needs and low levels of satisfaction with service providers in CaLD groups [8-10]. Evidence of this is found in the failure to provide early interventions or treatment to people with dementia or associated disorders, cultural clashes between caregivers and health professionals, and prejudicial attitudes and discrimination towards people with dementia from CaLD groups and their caregivers [11-13].

Ethno-specific aged care services, defined as a “*Service category based on ethnic, linguistic or religious community providing a service to its own members*” [14], are highly regarded by CaLD caregivers. However, this category of services differ markedly in terms of the range of available services, as some only have very minimal care services whilst others have a wider range of community aged care packages [12,15]. This is problematic given that caregivers who depend on bilingual care coordinators to use dementia services rely on the network of the care coordinators and their ability to facilitate cross-organisation collaboration in service delivery.

One key factor affecting the use of and satisfaction with dementia services of caregivers is the level of caregiver’s acculturation in the host culture [10,13,16]. Acculturation influences the social networks of caregivers and thus also shapes their ability to access information and resources in dementia care [17,18]. Berry [17] describes four levels of acculturation namely: assimilation, integration, isolation, and marginalization. Migrants can achieve assimilation if they give up their culture and adapt completely to the host culture. Most migrants are able to achieve integration, where they adopt their host culture whilst maintaining their own cultural values. However, migrants can become isolated if they are unable to interact with people from the majority culture and instead socialise with people from their own culture. Migrants are viewed as being marginalised if they have little contact with people from the majority culture or their own culture.

Communication barriers are also viewed as a major factor that inhibits CaLD caregivers to access, plan and manage dementia services to meet the care recipients’ and their own care needs [13,19]. However, most of the studies on this issue only explored this issue from the perspective of the service providers [12,13,20]. How the caregivers interact with care staff and whether they are able to comprehend the dementia care information are not clear. Communication barriers are further compounded by a lack of social networks for immigrants in a host country. To provide CaLD caregivers with the necessary social and emotional support, it is essential that social networks are established for them [18,21]. It should be noted that the NRCP provides caregivers with some social support; however, there are few studies that examine the experiences of CaLD caregivers with NRCP.

Another factor that shapes the use of dementia services by CaLD caregivers is the level of health literacy. Studies identified that the low health literacy of some CaLD family caregivers and a lack of ability to manage care tasks contributed significantly to the burden, depression, and lower quality of life for these caregivers [22,23]. Studies also trialled that dementia education programs for caregivers showed improved dementia care knowledge and a better ability to cope with care challenges [7,24,25]. In addition, well-designed caregiver interventions can also empower them to select and manage dementia services to meet the care recipients’ and their own quality of life needs [22]. The engagement of CaLD caregivers in dementia education programs and their satisfaction with the programs remains largely unknown because most studies in English-speaking countries exclude participants who are unable to speak English.

This study was undertaken to better understand the problems faced by CaLD caregivers in Australia and their utilisation of dementia services. Selected caregivers from Chinese, Greek, Italian and Vietnamese communities who cared for people with dementia were asked about their experiences when they utilised dementia services. These communities are representative of the Southern European and Asian cultures, and the immigration patterns in Australia [9,26].

### Aim

The aim of the study was to explore the experiences of family caregivers from Chinese, Greek, Italian and Vietnamese groups in utilising dementia services in Australia.

## Methods

### Design

Gadamer's philosophical hermeneutics was used to interpret the experiences of the participants. Gadamer believed that a human action or experience is similar to a “text” and the investigator must interpret this text [27]. The meanings derived from the interpretation of the text are a fusion of what the “actor” says and what it means to the interpreter in a socio-historical context. This philosophical tradition acknowledges that the interpreter has a prior understanding of the particular phenomenon under study and that the socio-historical context influences the interpretive processes.

### Setting

The study was conducted in community settings in a metropolitan city of Australia.

### Participants

A purposive sample of family caregivers was selected from four cultural groups, namely, Chinese, Greek, Italian, and Vietnamese. The selection criteria in the study included: (1) family caregivers who had at least one year of experience in caring for a person with dementia; (2) who had daily contact with the care recipient; and (3) resided in the same or a separate household in close proximity.

### Data collection and analysis

The major data collection methods were focus group discussions and face-to-face in-depth interviews with participants. Through group interactions, focus groups can generate a group synergy that is rarely generated in individual-based interviews [28]. However, individual-based interviews were also used if caregivers chose this type of participation or if they were unable to attend a scheduled focus group.

Data collection was conducted over a six month period in 2011. The focus group discussions or interviews were guided by a semi-structured discussion guide that asked questions of the participants in regard to the research aim. The guide was translated into four languages and printed out for the participants who were unable to speak English well or could not speak English at all. Five focus groups were conducted, each of which had two researchers with one acting as a facilitator and the other as the observer. The observer took notes on the group dynamics, which helped to provide the researchers with memory prompts during the analysis phase. To assist the participants who could not speak English, interpreters were used in the focus group discussions and interviews. The focus group discussions were conducted in the meeting rooms of community centres where the caregivers usually attended carer support group activities. The interviews were conducted in the homes of the participants. The focus group discussions were 120 to 150 minutes long, while the interviews lasted from 60 to 90 minutes. The focus group discussions and interviews were audio-recorded and transcribed verbatim for the data analysis.

A thematic analysis using the six phases recommended by Braun and Clarke [29] was undertaken. The first author undertook the initial data analysis and circulated the findings to the rest of the research team for cross-checking and discussion in scheduled meetings. During the preliminary analysis, each transcript was read for the descriptions were highlighted in the transcripts of the participants that were significant in terms of service utilisation. These highlighted descriptions were then coded manually. Once the coding was completed, the codes in the focus group discussions and interviews were compared to reduce the number of codes through clustering. The final codes were grouped and summarised based on their meaning into themes. The researchers then reviewed, discussed, and further refined the themes for the final research report based on the emphasis of importance and relevance according to the participants.

Study rigor was achieved by clarifying issues with the participants at the end of the focus group discussions or interviews, and by using sufficient excerpts from the participants to support the findings. Rigor was further enhanced by using interpreters, and by cross-checking the findings within the research team.

### Ethical considerations

The Social & Behavioural Research Ethics Committee of Flinders University approved the study (project No. 5008). Participants were contacted by letters that requested their voluntary participation in either a focus group discussion or an interview. Various community organisations were asked to help distribute the letters to potential participants. The letter enclosed a “participant information sheet”, a list of “semi-structured questions for focus group discussion and interview”, and a “participant’s response slip”. Participants who met the selection criteria and were willing to participate in a focus group discussion or an interview were asked to provide their contact details on the “participants response slip” and return it via a pre-paid, pre-addressed envelope. A researcher then contacted the participant by phone to arrange a time and venue for their participation in either a focus group discussion or interview. For those who chose to be interviewed, a researcher negotiated the interview time and venue with the participant. Caregivers in the study were adults over 18 years older and had capacity to give informed consent. The anonymity and confidentiality were discussed with participants and written informed consents were obtained from those caregivers prior to the focus groups or interviews.

## Results

In total, 46 family caregivers participated in the project. Of these, 39 and 7 were primary and secondary family caregivers respectively. These caregivers cared for 39 people with dementia in total. Tables 1 and 2 contain summaries of the demographic information of the persons with dementia and their caregivers.

**Table 1:** Demographic information of the person with dementia (n = 39)

**Items**	**Chinese**	**Greek**	**Italian**	**Vietnamese**
No. of persons with dementia	10	10	14	5
Male	1	7	4	1
Female	9	3	10	4
Age (range)	77.5 (72–90)	79.2 (75–86)	80.3 (75–94)	83.4 (78–89)
Years in Australia (range)	17.2 (6–27)	55.4 (51–63)	53.3 (28–61)	19.6 (17–26)
Years of dementia (range)	5.4 (2–8)	7.3 (2–10)	7.8 (3–11)	8.8 (6–16)

**Table 2:** Demographic information of family caregivers (n = 46)

**Items**	**Chinese**	**Greek**	**Italian**	**Vietnamese**
No. of caregivers (second caregiver)	13 (3)	10	17 (3)	6 (1)
Male	2	2	4	2
Female	11	8	13	4
Spouses	1	9	4	0
Children	10	1	13	5
Grandchildren	1	0	0	1
Siblings	1			
Age (range)	48.3 (35–85)	77.8 (52–83)	58.2 (47–84)	50.5 (18–58)
Born in Australia	0	1	12	0
Immigrated to Australia	13	9	5	6
Years in Australia for immigrants	19.3 (8–32)	51.2 (40–63)	53.2 (49–61)	6 (2–32)
Duration in years of a carer role (range)	5 (2–11)	7 (3–10)	7.8 (3–11)	7.1 (2–17)
Bilingual and bicultural knowledge	2	1	12	1
Cannot speak English well	11	9	5	5
Attended dementia care course	0	9	14	0
Did not attended dementia care course	13	1	3	6
Caregivers who lived in the same house	13	9	13	6
Did not live in the same house	0	1	4	0
Codes used in the excerpts	Chinese 1-13	Greek 1-10	Italian 1-17	Vietnamese 1-6

Analysis of the focus group discussions and interviews revealed four major themes regarding the experiences of the family caregivers in utilising community dementia services. These themes were: 1) negotiating services for the person with dementia; 2) the impact of acculturation on using the services; 3) the characteristics of satisfactory services; and 4) negative experiences in utilising services. The cross-cultural comparisons of the data revealed a number of similarities and also differences between the four CaLD groups within these themes.

### Negotiating services for the person with dementia

Although the participants were willing to care for their relatives, they also embraced the services that relieved them from burdensome care activities or substituted them when they were not available for the care recipients. However, they had expectations that services would be flexible to meet their schedules and were also culturally appropriate. Such expectations made the negotiation of services unique for the CaLD caregivers. The skills described by caregivers in negotiating services included planning, communication and the searching of services. An Italian daughter caregiver noted that she had to have good planning and negotiate skilfully with the service provider to substitute her caregiver role, as she stated in the following:

I work full time. I have to get up early in the morning. There are mornings where she doesn’t want to get up. Luckily, this morning she actually got up quite early, which was a saving grace. I have to shower her. I get her dressed. I have to take her to the toilet because sometimes she gets disoriented getting to the toilet. I get 15 hours home care all up for a whole week and I divide that up how I want it [Italian 3].

This daughter caregiver was a second generation member of an Australian Italian family and the community care organisation she relied on had well-established ethno-specific dementia services. Therefore, her negotiation with the services to suit the care needs of her mother and her timetable worked most of the time.

An interview with a Vietnamese daughter caregiver who belonged to the first generation of an Australian Vietnamese family and was unable to speak English well stated her ability to negotiate services was affected by her capacity to access information on available dementia services and her limited English proficiency. The mother of the caregiver had been diagnosed with dementia 15 years earlier and was in the advanced stage of dementia. However, the caregiver only used the low care package (CACP) via two service providers. The caregiver was not aware of available services for her when she needed them, as she stated in the following excerpt:

The XX [A service provider] helps me [by providing day care] two days a week … and the Domiciliary Care helps me one day a week. . . I don’t know what else to suggest and I feel they help me too much already. … I was admitted to a hospital to undergo surgery for bowel cancer and I did not know whom I should contact to help me care for mum. A social worker through the hospital arranged respite care in a nursing home for mum [Vietnamese 1].

This case suggests that service providers need to provide timely interventions in cases of care crises if the CaLD caregiver is unable to plan, negotiate and manage dementia care services independently. This study identified that newly established ethno-specific service providers could only offer very minimal services for the same cultural group compared with well-established ethno-specific service providers (see Table 3). In addition, caregivers with limited English may encounter great difficulties in comprehending the complex nature of community aged care services available. Therefore, it is crucial to prepare caregivers to gain better understanding through education and information sessions that are culturally and linguistically congruent for them and their circumstances.

**Table 3:** Ethno-specific care services available for the four groups

**Items**	**Chinese**	**Greek**	**Italian**	**Vietnamese**
Care support groups	√	√	√	√
Dementia care education	X	√	√	X
Day Care	√	√	√	√
Respite Care at Home	X	√	√	X
Respite Care in nursing homes	X	√	√	X
CACP^1^	X	√	√	√
EACH^2^	X	√	√	X
EACH-D^3^	X	√	√	X

^1^Community aged care package ^2^Extended aged care at home ^3^Extended aged care at home dementia.

An interview with a Chinese caregiver who was born overseas, but was educated in Australia revealed that her application for dementia services for her mother was affected by a lack of information and support at the clinic of a general practitioner when her mother was first diagnosed as she stated below:

I took her to the doctor and they basically tested her out – did a scan and then they said that yep she has dementia and it’s not going to get any better. . . . the doctors didn’t tell us anything about dementia care. I didn’t realise there were any services that were directly related to dementia. I learned about dementia care and found the day care service for mum through internet searches [Chinese 11].

Due to a lack of social networks and knowledge about healthcare services and the language barriers in the host country, the caregivers from CaLD backgrounds in our study relied on health professionals who provided the dementia diagnosis to gain information about dementia services and to refer them to the relevant service providers.

### The impact of acculturation on service use

In this study, most of the adult child caregivers in the Italian, Greek and Chinese groups showed an acculturation level of “integration”. By contrast, most of the spouse caregivers in all four CaLD groups and most of the adult child caregivers in the Vietnamese group demonstrated an acculturation level of “isolation”. An Italian adult child caregiver who was born in Australia described how she was able to explore services for her father by using her social network and bilingual language skills in the following excerpt:

The ones that I don’t use are the ones that don’t have ethno-specific paths. You want elderly people to be independent and in control of their own lives but the non-ethno-specific services make people dependent. People from a CaLD background don’t know how to navigate those services unless they’ve got children like me or people who know how to navigate. Elderly people don’t know how to navigate unless their English is fairly proficient [Italian 12].

Adult child caregivers who had been integrated into the host country demonstrated an ability to search for information about services and to negotiate with service providers, as a Greek caregiver stated below:

I am very aware of my parents’ culture. I organized services for them, talked to the care coordinator to make sure that the care staff understand my parents’ needs and also [to] make sure my parents are happy with these services [Greek 6].

The participants were also capable of monitoring the quality of care services and acting as an advocate on behalf of their parents, as one Italian caregiver stated:

I contacted the care coordinators when my mother had a fall in respite care. I believe the poor communication skills with clients among the staff contributed to the fall and I told the care coordinator that the staff there needed to improve their performance [Italian 7].

The caregivers revealed they sometimes differed from other family members in terms of their level of acculturation and social expectations regarding the care of relatives with dementia, as demonstrated in the following Chinese caregiver’s statement:

Although our culture expects that the first son will take care of the elderly parents, my older brother never did that for my parents. He and my other brothers only visit my parents occasionally. My mother lives with me. She cannot speak English and does not like to interact with strangers. They [the brothers] thought it was government’s responsibility to care for Mum. I couldn’t get help from them [Chinese 12].

Caregivers who mainly engaged with their own cultural groups and had limited contact with the majority culture relied on the CaLD community to provide information about dementia care. Dementia service utilisation for some participants relied on care coordinators, as a Greek caregiver explained:

When my husband showed memory loss, the care coordinator helped me to organise an appointment with a GP and to apply for care services. I have been in the care support group since his diagnosis of dementia. I have also received support from the care coordinator to apply for nursing home care recently after hospitalization for a fall [Greek 2].

Caregivers who were involved in well-established ethno-specific aged care organisations reported being able to gain timely support. However, newer immigrants who were cared for by community care organisations with very minimal services and a small number of bilingual staff may not be as well supported. A Chinese caregiver in her 80s who had multiple chronic conditions and was unable to speak English revealed those issues in the following:

They [the care coordinators] have done excellent work by providing respite care for my husband here and having me in the care support group. However, I am too old to care for my husband at home and need other care services. My children found services from other organisations for my husband [Chinese 5].

Lower levels of acculturation also affected the ability of the caregiver to engage in quality improvement of services, as stated by caregivers. One participant emphasised the filial piety of the Chinese culture, and another’s needs were not being met as the following excerpts highlight:

If the care staff forget to do something, I can always do it myself. You cannot complain considering that really, the care of an elderly parent is a family responsibility in my country [Chinese 2].

I stopped sending my father to the day care. I was told that he was a trouble-maker there. I have to keep him at home, even though he loves to interact with others [Vietnamese 3].

Thus, the different attitudes towards service providers, which were influenced by the level of acculturation, could affect the implementation and evaluation of dementia services in older and newly established CaLD communities. This indicates that not all members of Australian society will be able to obtain high quality dementia services unless the caregivers have been adequately prepared and informed, the service provider options have been improved, and independent evaluators have been employed to monitor the quality of care.

### The Characteristics of satisfactory services

The caregivers’ satisfaction with services was shaped by their expectations for culturally and linguistically congruent caregiver support. Under these expectations, the characteristics of satisfactory services described by the caregivers were: (1) supporting caregivers to develop ability to cope with daily care challenges; (2) providing timely interventions in cases of care crises; and (3) relieving the psychological-emotional burden of caregivers by providing caregiver support groups.

All caregivers in the study were eager to obtain dementia care knowledge and skills. The majority of caregivers in the Italian and Greek groups had attended dementia care courses that were translated into Italian and Greek through ethno-specific service providers. However, none of the Chinese or Vietnamese caregivers had attended similar dementia sessions as such courses were not available in their native tongue (see Tables 2 and 3). Focus group discussions about what dementia was and how to manage this chronic condition at home clearly identified the disparity between the groups in terms of their knowledge about dementia. This was exemplified by the statements of a Chinese caregiver who did not attend a dementia education program and an Italian caregiver who did attend a program:

Mum developed signs of forgetfulness and strange behaviours such as putting the electronic kettle on the stove to make the water boil. We blamed her and thought these strange behaviours were related to her older age. I contacted the GP when we thought she had gone mad when she accused her close friend of stealing her books and DVDs [Chinese 1].

Dementia could be the loved one repeating things, saying the same things over and over again. I would tell him a hundred times and then. I would feel really bad. After I attended the Alzheimer’s program for caregivers, I was starting telling Dad, “do what you like Dad, I don’t care”. I try not to get angry, rather, now I try to show Dad that I am caring for him and I speak without demeaning him. It’s about ensuring that he’s safe [Italian 3].

Even if they had attended a dementia education program, the caregivers found that the single program session that they attended when the care recipients were diagnosed with dementia did not provide them with enough knowledge or confidence to cope with the challenges they faced during their long caregiving journey. One way to obtain this knowledge and help was to call dementia service helplines. These resources were only accessed by those who knew about them and who were fluent in English, as an Italian caregiver described in the following:

I’ve also used a lot of Alzheimer Australia services. For example, I rang them up when she [the mother of the caregiver] started developing aggressive behaviours. Staff from the DBMAS [Dementia Behavioural Management Advisory Service] made several home visits to assess her when she was very aggressive. I was taught that her behaviour was caused by unmet needs and how to identify and meet her needs [Italian 4].

Some caregivers reported their ethno-specific service provider had appointed a bilingual care coordinator for the caregiver support group in the NRCP who made regular home visits to assess the needs of the caregivers. The care coordinator also functioned as a resource person who helped the caregivers use the services they needed. An example was an older Greek caregiver who was unable to speak English. Her husband had been discharged recently from hospital after prostate surgery. She described how she approached the care coordinator for help, as follows:

He was very resistant about taking medications, going to the toilet, washing and dressing. He was very angry each time when I tried to nurse him. He even tried to choke me by holding my neck. I felt hopeless and rang Des [the care coordinator]. She helped me to apply for new services that I have not used before, for example, behavioural management, nursing care for the wound, catheter, and continence services [Greek 1].

The timely crisis intervention of the bilingual care coordinator helped the transition to home care after hospitalization and was crucial in preventing a premature admission to residential aged care. This study showed that the majority of people with dementia had co-morbidities (see Table 1) and often had acute episodes of falls, urinary tract infections, chest infections, acute delirium, and behavioural changes. However, many caregivers had not engaged in culturally and logistically appropriate caregiver support groups and had no contact with a bilingual care coordinator. Instead, they had to cope with the care crises on their own, as one Chinese caregiver stated in the following excerpt:

One time mum got sick and she couldn’t walk. She had a fever and she was sitting in the dining room chair and she couldn’t get up at all. So she had to sit there all day and I couldn’t lift her up to get her to her room. I had to wait until my husband came home to help me lift her to the toilet and to her room so she could lie down. That’s when I realised that I could not handle this [Chinese 11].

The mother of this caregiver was soon admitted to a nursing home, even though the daughter was willing to care for her at home. Thus, because of the lack of timely support and interventions from service providers when they were needed, this caregiver did not use the EACH package to try to keep her mother at home longer.

Caregivers were attracted to care services if they were culturally and linguistically congruent, as a Chinese caregiver stated:

In the day care, they organised something that mum and dad used to love, namely, watching 1960s-1970s kind of movies. Yeah, they would love things like that and music and sometimes they have karaoke as well [Chinese 8].

Carer support groups played a key role in sharing information about different services. However, the usefulness of the information for the caregivers depended on the efforts of the bilingual coordinator to facilitate the care support group sessions, as a Greek caregiver explained:

George is incontinent and I did not know about free pads until the coordinator invited a past caregiver, Maria, to share her experience of caring for her mother. She told us about how to find information regarding free pads [Greek 4].

Most carer support groups were run by volunteers or the caregivers themselves, and the activities mainly focused on social and cultural activities such as cooking and sharing meals, and enjoying music and poetry. Although these sessions were also welcomed by the caregivers, the dementia care abilities of the caregivers were limited in this context of support. Formal education and information sessions were identified as being necessary to improve their caregiving skills.

### Negative experiences in utilising services

This study identified that the negative experiences of the caregivers when they utilised dementia services contributed to the irregular or short-term use of services, or complete withdrawal from needed services. The negative experiences described by CaLD caregivers were associated with cultural and linguistic factors. For example, An Italian caregiver attributed medication errors her mother experienced during respite care in a nursing home to inadequate intercultural communication that staff showed when caring for her mother who spoke little to no English. She had decided not to use the respite care again, as she explained:

I was on holiday and she was in there [a nursing home] for 2 weeks. They gave her someone else’s medication. So I wasn’t impressed and so that’s why I get a bit paranoid about sending her into respite. …it’s upsetting that such a mistake is made. The doctor said “That could be why she’s deteriorated so quickly, because she was given the wrong medication” [Italian 2].

Another Italian caregiver also shared her concern about intercultural communication in the healthcare system. She had not used respite care in a nursing home for her mother, even though she was entitled to apply for it:

My mother was admitted to a hospital. She had a fall as she did not know how to use the call bell or ask for help. I do not feel comfortable putting her in respite care in a nursing home. I have given up my holidays in order to care for her [Italian 8].

One Chinese caregiver was concerned about the loneliness her mother experienced in respite care in a nursing home because of the lack of culturally and linguistically appropriate care activities and social interactions.

She felt very isolated and had no one to talk to. It is better to keep her at home if there is no suitable place for her [Chinese 8].

The analysis also identified the negative experiences of the caregivers could come from the use of ethno-specific dementia services. For example, one Vietnamese caregiver was told not to send her father to the day care centre after he developed aggressive behavioural problems. She did not receive a referral from the service provider to a service that would help her to manage the behaviours. Therefore, she did not utilise any community care services and instead worked alone to care for her father at home:

I have to force him to take a bath. I have to remind him 2 to 3 days in advance and he gets angry. He challenges me when I need to give him a bath or his medication, and even a glass of water. He gets upset with me. Once he hit me when I went to give him his medication [Vietnamese 3].

Caregivers also expressed dissatisfaction with inflexible services that did not meet the care needs of people with dementia and their caregivers even when using ethno-specific services.

During the day, she [the mother of the caregiver] can look after dad. We have a problem in the evening. He's got what is called Sun-downers Syndrome, where he becomes very agitated. …He goes in and out of the home. …I requested services in the evening, but was told that no evening services were available for dad [Italian 2].

The underdevelopment of ethno-specific services in new immigrant groups, understaffing and lack of resources in these service providers might have contributed to the situations caregivers described. Supporting mechanisms, resource development and regulations could be strengthened when considering the establishment of ethno-specific dementia services appropriate to the needs of clients.

The negative experiences that these caregivers had when they utilised dementia services suggested that the service providers could be better prepared to meet the needs of CaLD groups. To facilitate the use of dementia services, it is also important to prepare caregivers to become informed participants who understand dementia, their rights and dementia service choices and flexibility.

## Discussion

This qualitative research revealed how caregivers interacted with service providers and their satisfaction and dissatisfaction with dementia services. There are very few studies that have performed similar in-depth analyses of CaLD caregivers’ perspectives of dementia services. Moreover, the few studies on this subject have tended to regard various CaLD groups as a uniform group. This may be inappropriate as it overlooks the diverse nature of different CaLD groups in terms of important factors such as their acculturation level and readiness to participate in planning and control of dementia services. Viewing various CaLD groups as a uniform group may also lead to inappropriate stereotyping that will prevent care service providers from understanding the needs of their clients [30].

In this study, the caregivers from the Vietnamese and Chinese groups generally praised the dementia services that they had received even though they may not have accessed the services they actually needed. Therefore, findings from this study suggest that caregivers need to be well-prepared as informed consumers in order to access or participate fully in the use and evaluation of care services, as has also been found in other studies [7,23]. Given the diverse nature of the different CaLD groups and the disparities of ethno-specific dementia services among the CaLD groups, future studies on the effectiveness of dementia services in Australian immigrant groups would be invaluable in planning of dementia care services.

The caregivers differed in their abilities to plan, negotiate and manage dementia services. The participants who could find the services through their social networks and information sources were generally also able to negotiate with service providers regarding the types of services needed and how they should be delivered. Such caregivers may only need information support while the more consumer-directed dementia care programs are being established in Australia [6,7]. The consumer-directed approach is likely to be suitable for caregivers who belong to the second generation of Italian and Greek families as this study revealed these caregivers had achieved assimilation to the Australian culture. They also possessed social networks and the ability to access information, unlike the Chinese and Vietnamese caregivers.

By contrast, the spouse caregivers in all four CaLD groups and the first generation of adult child caregivers in the Chinese and Vietnamese groups relied to some degree on care coordinators when utilising dementia services. This was because of the caregivers’ limited English and health literacy. In such cases, it may be advisable to allocate a service advisor who is also bilingual and not associated with the service providers to work with the caregivers in a one-on-one supportive manner. This approach would obviate the potential conflicts of interest between the consumer and the service provider that were identified in our study (see the section of “Negative experiences in utilising services”). In this study when conflicts of interest arose, the service providers were in a dominant position to decide on the solutions. The CaLD caregivers who had limited English and resources to cope with dementia caregiving were in a vulnerable position to negotiate services. Therefore, the use of a bilingual service advisor may better support this group of caregivers to negotiate services to meet the care recipient and caregivers’ needs.

It was also found that culturally and linguistically congruent dementia courses were not equally distributed between each CaLD group evidenced by the factor that none of Chinese and Vietnamese caregivers had attended any dementia education sessions. The lower level of participation of CaLD groups in planning, negotiating and managing dementia services may be a result of a lack of knowledge about dementia care and the range of dementia services. This study supports findings from other studies that family caregivers were usually underprepared for their role and that their education needs were largely overlooked by the health care system [31,32].

The dependence on care coordinators that some family caregivers exhibited when using dementia services may be caused by their limited health literacy. Most of the spouse caregivers in the Italian and Greek groups had a low level of literacy in their first language. These findings are consistent with previous studies in Australia that showed primary family caregivers were usually female, spouses of the cared-for person, had a lower level of educational qualifications, had lower incomes and were often aged 65 or over [2]. Similarly, studies in the United States of America and other countries found that approximately 30% of adults had limited health literacy that hampered their ability to access, comprehend health care information and to act as informed consumers when using care services and in managing chronic disease [23,33].

The CaLD caregivers may not be able to actively participate in utilising and monitoring dementia services unless they are well-prepared as informed consumers, know the service options, and can plan and manage dementia services based on updated knowledge of dementia care [7,15,23]. Given that caring for people with dementia is a long-term care journey and that CaLD caregivers differ in terms of literacy and learning needs, caregiver education should be individualised with regular updating of dementia knowledge, rather than being limited to single program sessions shortly after the diagnosis of dementia.

It should be noted that CaLD caregivers in this study generally come from a collectivist cultural background that values group achievements over individual ones, and family members in these cultures are often willing to make sacrifices in caring for the person with dementia [13,17]. Consequently, in CaLD groups family caregiving for people with dementia is typically shared by a variety of family members, whereas people with dementia in Anglo-Australian families are normally cared for by a spouse [2,12]. Collectivist cultures have shown some advantages in relieving dementia care burden via a shared care based on kinship [34,35]. However, such advantages cannot be overestimated if the immigrant family does not have kinship resources and an extended family in the adopted country. In addition, collectivist cultures are associated with unique family dynamics due to shared responsibility of care in the family and some of these dynamics, such as filial piety can place an additional burden on family caregivers [13,19]. If the family takes a collectivist caregiving approach, the whole family could be prepared as a unit so that all members can participate in dementia care and receive relevant and appropriate dementia education.

The current community aged care system in Australia is perceived as being complex and fragmented. This system requires caregivers to navigate and negotiate with different service providers [4,6,12]. The community aged care reform or the new community aged care system commenced in 2012 is trying to address these issues by simplifying low and high care in the form of “Home Care” packages and a “Behaviour Supplement” and by proposing the “Gateway” (a single entry point for accessing information) [6,15]. The new system is based on a consumer-directed dementia care model [7]. Although this model is viewed as giving more power and autonomy to consumers to plan and control dementia services, it may not be suitable for some CaLD groups who have very limited options to gain culturally and linguistically congruent dementia services due to the underdevelopment of ethno-specific aged care services in these groups. Experiences of CaLD caregivers in the new system will need to be examined in order to make the new model being implemented more attuned to the CaLD groups and their ethnic and language needs.

This study had several limitations. First, the participants were selected *via* CaLD community organisations. Thus, it remains possible that CaLD caregivers who do not use dementia services or who have no contact with CaLD community organisations may have different perspectives on dementia services. Second, the study did not include caregivers from the mainstream culture in the study. Therefore, the study is unable to distinguish whether the issues identified in this study are specific to CaLD caregivers or common to all caregivers of people with dementia. Further comparative studies between the CaLD caregivers and the mainstream cultural group are needed to examine these issues. Third, CaLD groups vary markedly within and across groups and/or in different social contexts. Thus, the findings of this study cannot be generalised, although the findings do identify similarities and also differences between the cultural groups may be transferable or applicable to other cultural groups not included in the study.

## Conclusions

This study reveals the experiences of CaLD caregivers in utilising dementia services and their satisfaction with these services. These experiences were strongly influenced by their ability to negotiate services, their language, ethnicity and acculturation levels. The caregivers’ judgment of dementia services was also influenced by their experiences and by limitations in their knowledge about dementia and the dementia services that were available. Sometimes, this led caregivers to have a high regard for service providers, even though these providers did not meet their care needs according to the participants themselves and Australian standards. The second generation of Italian and Greek adult child caregivers demonstrated independence in planning and managing dementia services, whereas the spouse caregivers in all groups and the first generation of Chinese and Vietnamese caregivers were somewhat dependent on care coordinators when utilising available services. Ethno-specific dementia services were not equally distributed in the CaLD groups in the study. Furthermore, the lack of intercultural understanding in dementia care and barriers in intercultural communication suggested that the newer immigrant groups found it more difficult to use or know about dementia services.

The present findings have several implications regarding the improvement of CaLD caregivers’ utilisation of dementia services. First, CaLD caregivers need to be fully informed of dementia services, dementia itself, their rights and their obligations in planning, utilising and managing the dementia services. This information needs to be given in a manner that is culturally and linguistically relevant to the caregivers. Second, the support mechanisms that help CaLD caregivers to make informed decisions need to be individualised, flexible and tailored to their needs. The findings indicate that CaLD caregivers are at different levels in terms of readiness in accessing, planning and managing dementia services. One-on-one sessions with a service advisor are ideal for those caregivers whose health literacy is limited and who are unable to communicate in English. Third, the evaluation of dementia services needs to involve different stakeholders, including independent evaluators. In addition, the evaluation of CaLD caregivers’ satisfaction with services needs be obtained in a culturally and linguistically congruent manner.

## Abbreviations

CaLD: Culturally and linguistically diverse; DBMAS: Dementia behavioural management advisory service; EACH: Extended community aged care at home; EACH-D: Extended community aged care dementia; NRCP: National respite for caregivers program.

## Competing interests

The authors declare that they have no competing interests.

## Authors’ contributions

LDX designed the research, developed study conception, collected, analysed and interpreted data, and developed the manuscript. ADB co-designed this research, developed study conception, collected, analysed and interpreted data, and made significant changes to the manuscript. LH and HK helped in the recruitment, data collection, data analysis and critiqued the manuscript. All authors read and approved the final manuscript.

## Pre-publication history

The pre-publication history for this paper can be accessed here:

http://www.biomedcentral.com/1472-6963/13/427/prepub
